# Moderating effects of humanistic care and socioeconomic status on the relationship among pain intensity, psychological factors, and psychological function in adults with cancer pain from a province of China: A cross-sectional study

**DOI:** 10.3389/fpsyt.2023.928727

**Published:** 2023-04-04

**Authors:** Shuyun Wang, Xuyan Wang, Xiaohong Liu, Chenxing Zhao, Jinju Duan

**Affiliations:** ^1^Department of Pharmacy, Second Hospital of Shanxi Medical University, Taiyuan, Shanxi, China; ^2^School of Traditional Chinese Medicine and Food Engineering, Shanxi University of Traditional Chinese Medicine, Taiyuan, Shanxi, China; ^3^Department of Pharmacy, Yangquan First People's Hospital, Yangquan, Shanxi, China; ^4^Department of Pharmacy, Linfen People's Hospital, Linfen, Shanxi, China

**Keywords:** humanistic care, cancer pain, psychological factors, psychological functions, pain intensity

## Abstract

**Objective:**

The objective of this study is to explore whether humanistic care practiced by clinical pharmacists and socioeconomic status moderate the associations among pain intensity, psychological factors (catastrophizing and resilience), and psychological function (depression and anxiety) in cancer patients with low levels of education and income in the Shanxi province in the Northwest of China.

**Methods:**

Our sample comprised 123 adult inpatients with cancer pain. Demographic variables were obtained from the Hospital Information System of The Second Hospital of Shanxi Medical University. Pain intensity, psychological factors, and psychological functions were evaluated with four scales, and humanistic care was practiced with a part of the patients by clinical pharmacists. First, univariate analyses were conducted, followed by moderating effect models.

**Results:**

The incidence of depression and anxiety in patients with cancer pain in our sample were 48.78 and 41.46%, respectively. Low levels of psychological resilience (63.37, SD 21.74) were in this study. Pain intensity was significantly associated with humanistic care and anxiety. Humanistic care practiced by clinical pharmacists moderated not only the association between resilience and pain intensity but also the association between pain intensity and anxiety. Education levels moderated the relationship between pain intensity and the psychological factors of catastrophizing and resilience. Income levels moderated the association between resilience and anxiety.

**Conclusion:**

Humanistic care is essential in moderating the association among pain intensity, psychological factors, and psychological functions in Chinese cancer patients, especially those from lower-level counties and rural areas. Furthermore, socioeconomic statuses, such as education level and income, cannot easily change quickly. Still, proper humanistic care can relieve pain more effectively, reminding us that medical staff should implement effective personalized interventions to reduce patients’ pain intensity.

## 1. Introduction

Pain is one of the most prevalent consequences of cancer, although increasing attention on the assessment and management of it. Pain prevalence rates were reported to be 55.0% during cancer treatment and 66.4% in the advanced stages of cancer (1). Recently, the International Association for the Study of Pain (IASP) revised the definition of pain that had been in use for 40 years, explaining that pain is a personal experience that is affected by biological, psychological, and social factors to varying degrees; people can perceive pain through life experience; and pain may have an impact on patients’ physical functions and social and mental health (2).

Sociodemographic factors have been extensively researched to identify trends within populations with chronic pain. In general, research has shown that African American individuals, those from rural and low-income communities, and individuals with lower levels of education and literacy are more vulnerable to the harmful effects of suffering (3, 4).

The existing literature (5–7) shows that pain catastrophizing, resilience, anxiety, and depression may affect individual pain perception and expression. Pain catastrophizing is a significant psychological factor involved in regulating behavioral responses to pain. It is defined as a belief system, coping strategy, and evaluation process when experiencing pain (6). Resilience can be defined as an individual’s ability to recover or “bounce back” from negative events and maintain their function (or even thrive and grow) in the face of ongoing stress (8). Aside from this, research has suggested that pain is related to mental health problems in patients with cancer, but the possible causation and direction of these associations are not clear (9, 10). The intensity of pain and the states of anxiety and depression also interact with each other; for example, the severity of depressive symptoms is associated with the frequency of pain complaints (11).

In addition, socioeconomic status could moderate the impact of psychological factors (catastrophizing and resilience) on pain intensity and psychological functions (depression and anxiety). A study in Nepal found that both pain intensity and income moderated the association between resilience and physical function in individuals with chronic pain, while income moderated the association between resilience, catastrophizing, and depression (6). Another study on a population of patients with chronic pain in rural Alabama indicated that age notably mediated the relationship between catastrophizing, depression, and pain (3). Robert et al. (12) also found that the relationship between catastrophizing and pain intensity was significantly moderated by education and social functioning in patients with rheumatoid arthritis in the United States.

Humanistic care involves a fundamental belief in the power of the care process to produce growth and change for people (13). Humanistic care can help patients to eliminate fear in multiple dimensions, improve their psychological threshold for pain, and become aware of pain control measures, thus enabling them to better cooperate with the treatment (14). Clinical pharmacists are professionals who are licensed pharmacists with specialized advanced training and provide patients with comprehensive drug management and related care in all medical areas (15, 16). Humanistic care is one of the intervention contents of clinical pharmacists. Pharmacist-led interventions have yielded excellent results and have been shown to play a positive role in many areas, such as when including pharmacists in cancer pain multidisciplinary management teams (17).

Humanistic care may moderate the relationship between pain intensity, psychological factors (catastrophizing and resilience), and psychological functions (depression and anxiety). Furthermore, most previous studies have focused on the moderating effect of socioeconomic status (e.g., education level and income), which cannot easily be changed in a short time, on the relationships among pain intensity, psychological factors, and psychological functioning.

In January 2018, three clinical pharmacists with professional qualifications in pain were assigned to the oncology department to provide multifaceted interventions for pain management, humanistic care is included in it. The multifaceted interventions included: (1) daily ward round: made ward rounds with the physician every day (working days only) to assess the patient’s pain, medication, and laboratory results, and advised the physician to determine the optimal drug treatment; (2) regular review of medical orders: checked each patient’s temporary and long-term medical orders and gave feedback and explanation of the problematic orders to the physician; and (3) humanistic care: humanistic care was defined as providing patients with necessary one-on-one and face-to-face medication guidance and education for patients when they are receptive and able to cooperate. To illustrate, when patients did not accept using opiates because of concerns about its addictive properties, the clinical pharmacists would tell patients that, with the correct use, addiction would not occur. When patients had a poor emotional state, the clinical pharmacists would talk with them and teach them some methods to change their perceptions. When patients struggled with the belief that their pain was uncontrollable, the clinical pharmacists would educate them that, with reasonable treatment, the pain could be relieved. Patients who do not accept or cannot cooperate were not given humane care.

In view of this fact, the objective of this study is to explore whether humanistic care practiced by clinical pharmacists and socioeconomic status moderate the association among pain intensity, psychological factors (catastrophizing and resilience), and psychological functions (depression and anxiety) in patients with cancer with low levels education and income in the Shanxi province in the Northwest of China. In 2020, China’s average *per capita* GDP value is 114,808 yuan, and Shanxi Province, with a *per capita* GDP of 50,528 yuan.

## 2. Research methods

### 2.1. Sample and setting

This was a cross-sectional study and was performed with a sample of inpatients with cancer pain between August 2018 and August 2021 at The Second Hospital of Shanxi Medical University, a 2,700-bed academic teaching hospital in Taiyuan, China. The sample size was estimated by the statistical calculation formula of a cross-sectional survey of related factors (5).

We included patients who met the following criteria: (1) hospital inpatients; (2) aged ≥ 18 years; (3) diagnosed with cancer; (4) conscious, could communicate independently, and could express their wishes clearly; (5) suffered from cancer pain for at least 1 week; (6) live in Taiyuan City or its surrounding areas, including county towns and rural areas.

The exclusion criteria were: (1) diagnosed with psychiatric or mental disorders, such as schizophrenia, bipolar disorder, and depression by the physician; (2) cognitive disorders; and (3) being unable to complete the questionnaires.

One clinical pharmacist recorded all these works. It is important to note that humane care, which was routine work only studied as a moderator, like socioeconomic status, not as an intervention in this study. Another clinical pharmacist identified potentially eligible patients by reviewing their medical records and psychiatric history. The eligible patients were first informed about the purpose and protocol of the study. Secondly, they verbally told consent to participate in the research if they agreed to participate; at the same time, they informed them that all information would be protected. For participants who could not read or write, the investigator read out the questionnaire items word by word without any further explanation and completed the questionnaires based on the patient’s responses.

The study adopted the 5th day of participants’ pain score and provided participants with questionnaires. The entire investigation may last 10–20 min. When they completed the questionnaires, investigators checked and asked participants to fill in any missing items.

### 2.2. Measures

Demographic variables were obtained from the Hospital Information System (HIS) of The Second Hospital of Shanxi Medical University. The variables of interest were age, gender, income, marital status, education level, living area, the primary site of cancer, degree of disease progression, and type of pain. Humanistic care, pain intensity, psychological factors, and psychological functions were evaluated by clinical pharmacists using five scales during the daily ward rounds of the multifaceted pharmacist-led guidance team.

#### 2.2.1. Pain intensity

The Faces Pain Rating Scale (FPS-R; IASP, 2001), used with permission from the IASP, is a self-reported pictorial scale that consists of six faces showing increasing levels of pain. The respondents are asked to select a face that best represents their level of pain at the time of assessment (2).

#### 2.2.2. Resilience

Psychological resilience was assessed using the Chinese version of the Conner and Davidson resilience scale (CD-RISC). The 25-item CD-RISC contains three subscales, namely tenacity (13 items), strength (8 items), and optimism (4 items). It is rated using a 5-point Likert scale from 0 (not true at all) to 4 (true all the time), with a total score of 0–100. Higher scores indicate higher levels of psychological resilience. The Cronbach’s α coefficient of the scale in the present study was 0.927 (18).

#### 2.2.3. Pain catastrophizing

The Chinese version of the Pain Catastrophizing Scale (PCS) was used to assess patient reports of catastrophic thinking. The 13-item scale asks respondents to rate the degree to which they have certain thoughts and feelings when experiencing pain using a 5-point Likert scale ranging from 0 (not at all) to 4 (all the time). The total score for overall catastrophizing is equal to the sum of the raw scores. Higher scores indicate greater levels of catastrophic thinking. The Cronbach’s *α* coefficient of the scale in the present study was 0.91 (19).

#### 2.2.4. Anxiety and depression

Anxiety and depression levels were assessed with the Hospital Anxiety and Depression Scale (HADS), which is a 14-item inventory used to examine the degree of anxiety and depression of patients in nonpsychiatric hospitals. The HADS has two subscales—the anxiety subscale (HADS-A) and depression subscale (HADS-D)—each consisting of seven items. A 4-point Likert scale (0–3) is used to rate the items. Higher scores represent more severe psychological distress. This instrument is widely used in clinical settings, and the Chinese version used in the current study has sound reliability, with a Cronbach’s alpha coefficient of 0.832. The Cronbach’s alpha coefficients of the HADS-A and HADS-D subscales were 0.753 and 0.764, respectively (20).

#### 2.2.5. Humanistic care

The humanistic care ability of clinical pharmacists was assessed with the Humanistic Care Scale (HCS), which is a 5-item to evaluate the humanistic care ability of clinical pharmacists by patients. This scale was referenced to the Watson Caritas Patient Score (WCPS). A 7-point Likert scale (1–7) is used to rate the items. The items empirically assess the patient’s subjective experience of receiving humanistic care. The items refer to such indicators as loving kindness, trust, dignity, a healing environment, and honoring beliefs and values. The total score ranged from 5 to 35, with higher scores indicating better humanistic care ability. The Cronbach’s α coefficient of the scale in the present study was 0.835.

### 2.3. Data analysis

All statistical analyses were performed using IBM SPSS 25.0 (IBM Corp., Armonk, NY, United States). Due to the methods of data collection, missing data were minimal, and thus, data imputations were not utilized in this analysis. Descriptive statistical analysis: the patients’ general demographic data and clinically relevant data were described by percentage.

Correlation Test of Social Factors, Humane Care (Independent Variable) and Pain Intensity, Psychological Factors and Psychological Function (Dependent Variable): When the dependent variable is a continuous variable, the independent variable is categorical, One-Way ANOVA (multivariate variable) and t-test (binary variable) are used.

Correlation test between Pain Intensity, Psychological Factors, and Psychological Function: (1) taking pain intensity as the dependent variable and psychological factors as the independent variable; (2) taking pain intensity as the dependent variable and psychological function as the independent variable; (3) taking a psychological function as the dependent variable and psychological factors as the independent variable, using Pearson correlation analysis or Spearman correlation analysis.

Moderating effect tests: (1) we performed moderating effect tests of socioeconomic status on the relationship among pain intensity, psychological factors, and psychological functioning with hierarchical regression analysis. We used pain intensity and anxiety as the dependent variables. Subsequently, we performed moderating effect tests of socioeconomic status on the relationship between pain intensity, psychological factors, and psychological functioning. In the first step, we entered anxiety when testing pain intensity and entered pain intensity when testing anxiety to control the potential confounding effects on both the predictor and criterion variables. In the second step, we entered the socioeconomic variables of education level and income. In the third step, we entered the psychological variables of pain catastrophizing and resilience. In the fourth step, we entered the 12 interaction terms representing income × anxiety, income × depression, income × catastrophizing, income × resilience interaction effects, education × anxiety, education × depression, education × catastrophizing, and education × resilience interaction effects stepwise. (2) we performed moderating effect tests of humanistic care on the relationship between pain intensity, psychological factors, and psychological functioning. In the first step, we entered humanistic care. In the second step, we entered the psychological variables of pain catastrophizing and resilience. In the third step, we entered five interaction terms representing humanistic care × catastrophizing, humanistic care × resilience, humanistic care × pain intensity, humanistic care × anxiety, and humanistic care × depression interaction effects stepwise. Statistical significance was set at the level of 0.05 or less (two-tailed).

Outliers and missing data were not found in our study. All variables were normally distributed. The data met the necessary hierarchical regression analysis.

The study was approved by the research ethics committee of The Second Hospital of Shanxi Medical University (2021–242).

## 3. Results

### 3.1. Participant attributes

We enrolled 51 male and 72 female patients in the study (*N* = 123). Their average age was 56.26 years, with an SD of 19.09 years. More than half of the patients had 6 years of education or less (*n* = 69, 56.1%). Most of the participants (*n* = 120, 97.6%) had medical insurance. The highest incidence of carcinoma was chest tumors (*n* = 37, 30.1%), followed by abdominal tumors (*n* = 29, 23.6%). In total, 65.0% of the patients were locally advanced, and 61.8% of them were suffering from mixed pain. The demographic and clinical characteristics are summarized in Table 1.

**Table 1:** Description of the study sample (*N* = 123).

Characteristic	*N* (%)
Age (years)	
≤ 50	33 (26.8)
51–70	63 (51.2)
> 70	27 (22.0)
Sex	
Male	51 (41.5)
Female	72 (58.5)
Educational level	
None	9 (7.3)
Primary/below (≤ 6 years)	60 (48.8)
Middle (7–12 years)	39 (31.7)
High	15 (12.2)
Income	
0	9 (7.3)
≤ 1,000	51 (41.5)
1,000–3,000	45 (36.6)
≥ 3,000	18 (14.6)
Medical insurance type	
Provincial/city insurance	105 (85.4)
Resident health insurance	15 (12.2)
Own expense	3 (2.4)
Living area	
City (TaiYuan)	38 (30.9)
County seat	33 (26.8)
Rural area	52 (42.3)
Primary cancer site	
Abdominal tumor	29 (23.6)
Urinary tumor	3 (2.4)
Chest tumor	37 (30.1)
Cervical cancer	15 (12.2)
Osteosarcoma	12 (9.8)
Leukemia and lymphoma	3 (2.4)
Head and neck	11 (8.9)
Breast cancer	13 (10.6)
Extent of disease	
First stage of cancer	43 (35.0)
Locally advanced	80 (65.0)
Type of pain	
Nociceptive pain	44 (35.8)
Neuropathic pain	3 (2.4)
Mixed pain	76 (61.8)

### 3.2. The correlation between variables.

The univariate correlations among the study variables are presented in Tables 2, 3. As can be seen, education, sex, and age in years were not significantly related to any of the standard variables. Humanistic care was significantly related to depression and marginally statistically associated with anxiety and pain intensity. Income had a significant correlation with resilience (*p* < 0.05). Anxiety levels showed a statistically significant moderate positive correlation with pain intensity (*r* = 0.361, *p* < 0.05). There was a statistically significant moderate negative correlation between both anxiety and depression and resilience (*r* = −0.346, *p* < 0.05 and *r* = −0.423, *p* < 0.01, respectively). Catastrophizing showed a statistically significant moderate negative correlation with resilience (*r* = −0.435, *p* < 0.01). There was a statistically significant strong positive correlation between both anxiety and depression and catastrophizing (*r* = 0.702, *p* < 0.01 and *r* = 0.597, *p* < 0.01, respectively).

**Table 2:** The *p*-value of comparisons between categorical variables.

	Catastrophizing	Anxiety	Depression	Resilience	Pain intensity
Educational level	0.209	0.216	0.418	0.774	0.347
Income	0.155	0.115	0.157	**0.035**	0.350
Age (years)	0.630	0.556	0.846	0.075	0.491
Sex	0.750	0.529	0.498	0.135	0.274
Humanistic care	0.479	0.061	**0.043**	0.478	0.059

When *p* < 0.05, the values have been highlighted in bold.

**Table 3:** Mean and SD values of the continuous variables and correlation coefficients between the continuous variables.

	Mean ± SD	Catastrophizing	Anxiety	Depression	Resilience	Pain intensity
Catastrophizing	26.61 ± 13.32		0.702^***^	0.597^***^	−0.435^***^	0.293
Anxiety	7.54 ± 4.89				−0.346^**^	0.361^**^
Depression	7.85 ± 5.30				−0.423^***^	0.138
Resilience	63.37 ± 21.74					−0.172

^**^p < 0.05 and ^***^p < 0.01.

### 3.3. Moderating effects of humanistic care and socioeconomic status on the relationship among pain intensity and psychological factors and psychological function

The results of the moderating effect test are presented in Tables 4, 5. In the first step, anxiety made a statistically significant contribution to pain intensity, and pain intensity made a statistically significant contribution to anxiety. As can be seen, education moderated the associations of resilience and pain catastrophizing with pain intensity. Pain intensity and depression moderated the association of pain catastrophizing with anxiety, and income moderated the association between resilience and anxiety (Table 4). Furthermore, humanistic care moderated not only the association between resilience and pain intensity but also the association between pain intensity and anxiety (Table 5).

**Table 4:** The moderating effect of socioeconomic status on the relationship among pain intensity, psychological factors, and psychological functioning.

	Total *R*^2^	Δ*R*^2^	F-Δ*R*^2^	Standardized beta coefficient (B)	*p*-Value
Pain Intensity as the criterion variable, Educational Level*Resilience as the interaction term					
	0.147	0.147	6.591		
Anxiety				0.084	0.694
	0.220	0.073	3.412		
Income				0.176	0.303
Education level				−1.812	0.008
	0.222	0.002	1.994		
Resilience				−1.547	0.339
Catastrophizing				0.190	0.003
	0.380	0.158	3.311		
Educational Level*Resilience				2.511	0.002
Pain Intensity as the criterion variable, Educational Level*Catastrophizing as the interaction term					
	0.147	0.147	6.591		
Anxiety				0.322	0.118
	0.220	0.073	3.412		
Income				0.167	0.338
Education level				1.207	0.003
	0.222	0.002	1.994		
Resilience				1.273	0.009
Catastrophizing				−0.005	0.974
	0.408	0.186	3.909		
Educational Level*Catastrophizing				−1.627	0.006
Anxiety as the criterion variable, Income*Resilience as the interaction term					
	0.147	0.147	6.591		
Pain Intensity				0.224	0.047
	0.275	0.127	4.543		
Income				0.147	0.227
Education level				−1.033	0.001
	0.575	0.301	9.177		
Resilience				−0.779	0.005
Catastrophizing				0.564	0.000
	0.672	0.097	11.523		
Income*Resilience				1.300	0.003

**Table 5:** The moderating effect of humanistic care on the relationship among pain intensity, psychological factors, and psychological functioning.

	Total *R*^2^	Δ*R*^2^	F-Δ*R*^2^	Standardized beta coefficient(B)	*p*-Value
Pain Intensity as the criterion variable, Humanistic care*Resilience as the interaction term					
	0.147	0.147	6.591		
Anxiety				0.405	0.092
	0.150	0.002	3.339		
Humanistic care				−1.096	0.071
	0.151	0.001	1.594		
Resilience				−1.048	0.045
Catastrophizing				−0.077	0.734
	0.253	0.102	2.265		
Educational Level*Resilience				1.307	0.035
Anxiety as the criterion variable, Humanistic care*Pain Intensity as the interaction term					
	0.147	0.147	6.591		
Anxiety				−0.828	0.011
	0.560	0.413	23.771		
Humanistic care				−0.464	0.122
	0.627	0.067	15.055		
Resilience				−0.130	0.210
Catastrophizing				0.359	0.008
	0.718	0.092	17.770		
Humanistic care*Pain Intensity				1.519	0.002

## 4. Discussion

The key finding from this study was that humanistic care practiced by clinical pharmacists moderated the associations among pain intensity, psychological factors, and psychological functions, which has rarely been studied previously. From another perspective, these findings suggest that pharmacist-led interventions play a positive role in cancer pain multidisciplinary management teams.

The frequencies of depression and anxiety are higher in cancer patients, but prevalence rates vary greatly between studies. In patients with cancer, estimated prevalence rates range between 11 and 57% for depression and between 6.5 and 23% for anxiety (21, 22). The results of our study showed that the incidence of depression in patients with cancer pain was 48.78%, within the range of previous literature reports. However, the incidence of anxiety was 41.46%, which is higher than the previously reported range. Naser et al. (22) found that anxious symptomatology was more prevalent in patients with lung cancer in inpatient settings. Similarly, the most common cancer type in our study was lung cancer (27.9%). Additionally, the frequency of depression was higher than anxiety in our study, which is consistent with other studies (23, 24). Patients who were in advanced disease stages were particularly susceptible to suffering from depression, and 65.9% of our patients were in advanced disease stages. Our study reported a low level of psychological resilience (63.37 ± 21.74), which was similar to the level found in Chinese cancer patients in a previous study (65.46 ± 13.93) (25). Low resilience is linked to mood disorders (18), and this may, thus, be a reason for the high rates of anxiety and depression detected in our sample.

Through a univariate analysis, we found that pain intensity was notably associated with anxiety. Unseld et al. (21) highlighted that most studies suggest that depression may be more frequently related to pain than anxiety, but the results are controversial. The possible reason for pain intensity being associated with anxiety in this study is that our sample included a wide range of cancer types, while the samples of those previously reported studies focused on specific cancer types, such as colorectal cancer, breast cancer, or lung cancer.

Pain catastrophizing is considered one of the most important modifiable psychosocial predictors of pain intensity (26). Our analysis revealed that pain catastrophizing was not notably associated with pain intensity, which is inconsistent with prospective studies (27), which have found that pain catastrophizing is a robust predictor of greater pain severity. However, other studies also highlight that, although pain catastrophizing is commonly associated with pain intensity, there is limited evidence showing that changes in pain catastrophizing causes changes in pain (26, 28). Rizzo et al. (26) performed longitudinal assessments for the mediating effect of pain catastrophizing on pain intensity and drew the conclusion that the timing of the assessment influenced the mediating role of pain catastrophizing on pain intensity. However, we did not conduct the self-report measures of pain catastrophizing with patients at a fixed time because of the absence of patients when we made ward rounds. This may explain why pain catastrophizing was not notably associated with pain intensity in our investigation.

The results of the moderating effect test showed that neither pain catastrophizing nor resilience made statistically significant independent contributions to the prediction of pain intensity. However, when adding the moderator of education level, both pain catastrophizing and resilience had statistically significant relationships with pain intensity. Importantly, the finding that education level moderated the relationship between pain catastrophizing and pain intensity is consistent with previous studies, which found that high pain catastrophizing was linked to low education, which, in turn, led to inappropriate pain-coping strategies (29). Indeed, Cano et al. suggested that numerous pain-coping strategies, such as the ability to distract and reinterpret, may rely on cognitive skills that are potentially enhanced by higher education and primary literacy (3). Individuals with lower levels of literacy may have fewer cognitive resources available to navigate the management of chronic pain, thus increasing the risk for distress and negative thinking patterns and ultimately exacerbating the pain condition (3). Furthermore, cognitive flexibility is reported to be a critical factor in preventing negative outcomes and suicidal behavior in response to stressful life events (30). Overall, individuals with high levels of literacy may have more resources available to cope with stress and the burden of illness (18, 31). When patients with chronic diseases have higher mental resilience, they show higher degrees of acceptance of the disease, higher compliance with the treatment plan, and better prognoses (18, 32).

Regarding depression and anxiety, depression has received more attention from researchers, and its adverse effects on physical functioning and quality of life are well established (33). However, we chose to discuss anxiety, which has been studied less frequently, as a predictor of pain intensity, because pain intensity was not significantly associated with depression in the current study. The results of this study indicated that higher income contributed to a higher level of psychological resilience in patients with cancer pain, which supports the theory proposed by Wister et al. (34), and income significantly moderated the association between resilience and anxiety (income × resilience interaction; *β* = 1.300, *p* = 0.003). These data are consistent with reports describing the prediction of depression. However, income was not significantly associated with anxiety in our investigation, which may have resulted from the fact that nearly half of the sample were unemployed or farmers, whose incomes are at low levels; indeed, such drastic poverty may function as a leveling factor (29). People with low incomes experience negative emotions, which in turn affect resilience levels (35).

More importantly, considering the moderators of education level and income cannot be changed easily in a short time, we further investigated the moderating effect of humanistic care. In the present investigation, humanistic care practiced by clinical pharmacists moderated not only the association between resilience and pain intensity but also the association between pain intensity and anxiety. This suggests that, with patients with low socioeconomic status, medical staff should focus more on humanistic care to reduce their negative emotions and relieve their pain intensity. A previous study suggested that health knowledge education could work in the short term, especially when patients were seriously ill or had severe pain (14). Additionally, Edwards et al. confirmed that pharmacist educational interventions for cancer pain patients showed promise in reducing pain intensity (36). A number of publications have indicated that the multifaceted pharmacist-led guidance team intervention successfully decreases drug-related problems and shows both initial and prolonged pain relief (37). In summary, humanistic care practiced by clinic pharmacists could improve patients’ awareness of cancer pain to enable them to overcome their fears and build confidence, thus making pain management more humanized, scientific, and comprehensive to effectively relieve pain.

### 4.1. Study limitations

The findings of the current study have a number of limitations that should be considered when interpreting the results. Firstly, this study used cross-sectional data, which limits the conclusions that can be drawn with respect to causal relationships. The underlying reasons for the associations found in the present analyses remain to be fully understood. It is possible to use longitudinal measurements to examine the relationship between mediator and outcome variables and allow inferences of causality in further research. Secondly, the sample’s demographic homogeneity is a potential limitation; to determine whether rurality itself is a predictor of poorer pain outcomes, it would be important to compare the findings of this rural population with low socioeconomic status to those of an urban population with similar demographic features. Thirdly, the sample was obtained from a single institution during a limited study period, and, thus, the results may not be widely representative or generalizable.

### 4.2. Clinical implications

Our research emphasizes the importance of humanistic care practiced by clinical pharmacists for patients with cancer and low levels of education and income in the Northwest of China. Clinical pharmacists could better provide patients with cancer pain with cognitive resources to reduce their negative thoughts and improve their awareness in order to overcome fear, build confidence, and increase their mental resilience in a short time. Furthermore, this would improve their acceptance of pain, enhance their compliance with treatment plans, and enhance the therapeutic effects.

Additionally, the results of this study highlight the need to pay more attention to screening for psychiatric disorders, such as depression and anxiety, in inpatients with cancer pain. To optimize treatment, a positive screening result should be followed by a thorough psychiatric diagnostic interview conducted face-to-face. Therefore, adequate pain-related treatment should be discussed by a multidisciplinary team, which may include doctors, clinical pharmacists, and nurses.

## 5. Conclusion

This study found that humanistic care plays an important role in moderating the associations among pain intensity, psychological factors, and psychological functions in Chinese patients with cancer, especially for those from counties and rural areas with lower levels of income. From another perspective, this study shows that pharmacist-led interventions play a positive role in cancer pain multidisciplinary management teams.

Furthermore, in this study, there was a high incidence of both anxiety and depression, and pain intensity was significantly associated with humanistic care and anxiety. After adjusting for these associations, the results showed that education levels moderate the relationship between pain intensity and both pain catastrophizing and resilience. Additionally, income moderates the relationship between resilience and anxiety.

## Data availability statement

The original contributions presented in the study are included in the article/Supplementary material, further inquiries can be directed to the corresponding author.

## Ethics statement

The studies involving human participants were reviewed and approved by The research ethics committee of the Second Hospital of Shanxi Medical University. The patients/participants provided their written informed consent to participate in this study.

## Author contributions

SW, XW, XL, and CZ conducted the literature review and did the statistical analysis. SW, XW, XL, CZ, and JD contributed to the interpretation of data and to the study concept and design. SW, XL, and JD contributed to drafting the paper. All authors contributed to the article and approved the submitted version.

## Funding

This study was supported by the Second Hospital of Shanxi Medical University Science Foundation (Grant number 202202-7) and Shanxi Provincial Health Commission Science Foundation (Grant number 2019118).

## Conflict of interest

The authors declare that the research was conducted in the absence of any commercial or financial relationships that could be construed as a potential conflict of interest.

## Publisher’s note

All claims expressed in this article are solely those of the authors and do not necessarily represent those of their affiliated organizations, or those of the publisher, the editors and the reviewers. Any product that may be evaluated in this article, or claim that may be made by its manufacturer, is not guaranteed or endorsed by the publisher.
